# The Transmissibility of Antibiotic-Resistant Enterobacteriaceae in Intensive Care Units

**DOI:** 10.1093/cid/cix825

**Published:** 2017-09-15

**Authors:** Tanya Gurieva, Mirjam J D Dautzenberg, Marek Gniadkowski, Lennie P G Derde, Marc J M Bonten, Martin C J Bootsma

**Affiliations:** 1 Julius Center for Health Sciences and Primary Care; 2 Department of Medical Microbiology, University Medical Center Utrecht, The Netherlands; 3 Department of Molecular Microbiology, National Medicines Institute, Warsaw, Poland; 4 Department of Intensive Care Medicine, University Medical Center Utrecht; 5 Faculty of Sciences, Department of Mathematics, Utrecht University, The Netherlands

**Keywords:** ESBL, transmission capacity, *E. coli*, *K. pneumoniae*, reproduction number

## Abstract

**Background:**

The global emergence of infections caused by Enterobacteriaceae resistant to expanded-spectrum cephalosporins (ESCs) in intensive care units (ICUs) is, at least partly, driven by cross-transmission. Yet, individual transmission capacities of bacterial species have not been quantified.

**Methods:**

In this post hoc analysis of a multicenter study in 13 European ICUs, prospective surveillance data and a mathematical model were used to estimate transmission capacities and single-admission reproduction numbers (*R*_*A*_) of *Escherichia coli* and non–*E. coli* Enterobacteriaceae (non-EcE), all being ESC resistant. Surveillance was based on a chromogenic selective medium for ESC-resistant Enterobacteriaceae, allowing identification of *E. coli* and of *Klebsiella*, *Enterobacter*, *Serratia*, and *Citrobacter* species, grouped as non-EcE.

**Results:**

Among 11420 patients included, the admission prevalence was 3.8% for non-EcE (74% being *Klebsiella pneumoniae*) and 3.3% for *E. coli*. Acquisition rates were 7.4 and 2.6 per 100 admissions at risk for non-EcE and *E. coli*, respectively. The estimated transmission capacity of non-EcE was 3.7 (95% credibility interval [CrI], 1.4–11.3) times higher than that of *E. coli*, yielding single-admission reproduction numbers (*R*_*A*_) of 0.17 (95% CrI, .094–.29) for non-EcE and 0.047 (95% CrI, .018–.098) for *E. coli*.

**Conclusions:**

In ICUs, non-EcE, mainly *K. pneumonia*e, are 3.7 times more transmissible than *E. coli*. Estimated *R*_*A*_ values of these bacteria were below the critical threshold of 1, suggesting that in these ICUs outbreaks typically remain small with current infection control policies.

## Teaser

In an international study in 13 European intensive care units using protocolized surveillance and microbiological analysis, the transmission capacity of bacteria resistant to expanded-spectrum cephalosporins was 3.7 times higher for non-*Escherichia coli* Enterobacteriaceae (mainly *Klebsiella pneumoniae*) than for *E. coli.*

**See the Editorial Commentary by Birgand et al on pages 494–6.)**

Incidences of infections caused by Enterobacteriaceae resistant to expanded-spectrum cephalosporins (ESCs) have increased in the last decade, especially in intensive care units (ICUs) [1, 2]. Most infections are preceded by asymptomatic carriage, especially in the intestine, which may not be apparent at the time of ICU admission. ICU-acquired colonization with these bacteria may originate from an exogenous source, for instance through patient-to-patient transfer of bacteria, from horizontal transfer of resistance genes located on mobile genetic elements, or from within-host selection of previously undetectable bacteria.

In recent years, the understanding of the epidemiology of ESC-resistant Enterobacteriaceae in ICUs has increased. For instance, several studies have demonstrated that spontaneous decolonization during ICU stay is rare, as about 80% of the carriers of extended-spectrum β-lactamase (ESBL) genes were still colonized after 1 month [3]. Yet, other quantities, such as horizontal gene transfer rates, have not been determined, and it is unknown whether there is heterogeneity in transmission potential of different gram-negative bacteria. Quantification of these parameters is essential for understanding the transmission dynamics and, hence, for the design of effective infection control measures in ICUs.

Yet, estimation of the relative importance of the different acquisition routes is complex. The main difficulty is that the exact timing of acquisition of bacteria cannot be demonstrated accurately. Clinical culture results will miss many episodes of carriage, as carriage infrequently leads to infection. Results from regularly obtained surveillance cultures are more useful, but even these suffer from limited sensitivity and interval censoring, as screening cultures are collected at discrete time points precluding determination of the exact times of acquisition. Recent work, though, has provided statistical methods to better address this problem [4–6].

Here we have used screening culture results from 11420 patients during 48 months (122301 patient-days) in 13 European ICUs that participated in the mastering hospital antibiotic resistance, a cluster randomized intervention study in intensive care units (MOSAR-ICU) trial [7]. Screening on a chromogenic selective medium, followed by microbiological analysis, distinguished *Escherichia coli* from other bacteria, such as *Klebsiella*, *Enterobacter*, *Serratia*, or *Citrobacter* species, here grouped as non–*E. coli* Enterobacteriaceae (non-EcE). We used a Bayesian random-effects method [6] to estimate the transmission capacity for *E. coli* and non–*E. coli* Enterobacteriaceae, and confirmed the findings with typing by the Raman spectroscopic analysis.

## METHODS

### Setting and Patients

Our analyses are based on the detailed data and molecular characterization of isolates from the MOSAR-ICU trial [7], a study in 13 ICUs in 8 European countries carried out between May 2008 and April 2011. This consisted of a 6-month baseline period, followed by a 6-month period in which a hand hygiene improvement program was implemented in combination with chlorhexidine body washing of all patients. Finally, ICUs were cluster-randomized to different approaches of screening and isolation of carriers of antibiotic-resistant bacteria. In all 3 study periods, carriage with ESC-resistant Enterobacteriaceae was determined on admission and twice weekly by obtaining perianal swabs. In the first and second period, there was no feedback of screening results to physicians, precluding adaptation of infection prevention measures. In the third study period, screening was followed by contact precautions for identified carriers. Patients aged ≥18 years with an expected length of stay of ≥3 days, and a sample of patients with shorter expected length of stay were included. There was no statistically significant effect of any of the interventions on acquisition rates of ESC-resistant Enterobacteriaceae [7].

### Microbiological Analysis

Swabs were plated onto the Brilliance ESBL 2 Agar (Oxoid Ltd, Cambridge, United Kingdom) and colonies from the groups of *E. coli* and *Klebsiella*/*Enterobacter*/*Serratia*/*Citrobacter* (non-EcE) were selected.

One colony of each morphotype per patient was frozen and transported to the National Medicines Institute, Warsaw, Poland, for further analysis. The Vitek 2 system (bioMérieux, Marcy l’Etoile, France) was used for species identification, followed by more specific analyses of Enterobacteriaceae with ESBL-, AmpC-type cephalosporinase–, or carbapenemase-mediated phenotypes of ESC resistance, as described previously [8–10].

### Mathematical Modeling

We assumed that the risk for an uncolonized patient per day to acquire colonization equals $\;\alpha +\beta \frac{I(t)}{N(t)}$. The term $\beta \frac{I(t)}{N(t)}$ describes the rate of cross-transmission, where *N(t*) is the total number of patients present in ICU at day *t* and *I(t*) is the number of colonized patients in the ICU at day *t*. The constant term *α* represents the risk of acquisition due to all routes, which do not depend on the number of colonized patients present in the ICU. This includes transmission due to visitors or persistently colonized healthcare workers, de novo mutations in the patient, and outgrowth of previously undetectable colonization, stimulated by antimicrobial use. The parameter *β* represents the effective transmissibility of the bacteria in the ICU, taking infection control measures such as hand hygiene into account. The effective single admission reproduction number *R*_*A*_, defined as the average number of secondary cases per primary case during the ICU admission of the index case when all other patients in the ICU are susceptible for acquisition [11, 12], is approximately *β<LOS>*, with *<LOS>* the mean length of stay of patients in the ICU. Note that secondary cases may remain undetected when they are discharged before samples for microbiological testing have been obtained, and that secondary cases may be detected after discharge of the index case. Tertiary cases (infected by secondary cases) are not part of the definition of *R*_*A*_.

We estimated the parameters *α* and *β* for *E. coli* and non-EcE (as a group) and the relative cross-transmission capacity of non-EcE vs *E. coli* (*β*^*non-EcE*^*/β*^*E. coli*^), using data on the days of admission, days of discharge of patients, the surveillance culture dates, and culture results. For simplification, we assume that the specificity and sensitivity of the tests were 100% (see Supplementary Material 3 for a discussion of the impact of this assumption). We assumed that colonization with *E. coli* does not change the hazard rate to acquire non-EcE colonization (and vice versa) and that colonization does not disappear during ICU stay (which seems reasonable, as the length of ICU stay is short and antibiotic pressure in ICU is high). We calculated the likelihood of values of *α* and *β* by averaging over all possible transmission paths that are in agreement with the culture results. For more details about the method, see Supplementary Methods and Bootsma et al [6]. In this way, we obtained, for both *E. coli* and non-EcE, the likelihood of the transmission parameters *α* and *β* per study period and per ICU on an (*α*, *β*) grid.

Because of the uncertainty levels around the transmission parameters per period and per ICU, results of 13 ICUs were pooled using a random-effects model as ICUs differ in many aspects (such as bed occupancy, admission prevalence, case mix, and adherence to infection prevention measures). We assumed that the parameters *α* and *β* in a single ICU are drawn from 2 independent folded normal distributions with means *α*_*0*_ and *β*_*0*_ and standard deviations *σ*_*α*_ and *σ*_*β*_, all with uninformative priors. Using the likelihoods obtained in the previous step, we obtained a posterior probability density for each point on the (*α*_*0*_, *β*_*0*_) grid by numerical integration over all possible values of the transmission parameters in each ICU (see Supplementary Material 2). In performing these analyses for non-EcE species and *E. coli*, we determined both the transmission parameters and the relative cross-transmission capacity of *E. coli* and non-EcE with the highest posterior probability. As the studied interventions did not reduce acquisition with ESC-resistant Enterobacteriaceae [7], we assumed in our primary random-effects analysis that transmission parameters within an ICU did not change between study periods. In the Supplementary Materials, we also present results per period and for a scenario in which interventions may have had an effect on transmission. In contrast to Derde et al [7], we base our estimates of the admission prevalence only on patients for whom a swab was obtained within 2 days after admission. This leads to slightly higher estimates of the admission prevalence.

### Raman Spectroscopic Analysis

To confirm differences in cross-transmission rates, we further typed relevant *K. pneumoniae* and *E. coli* isolates from ICUs with at least 25 patients being colonized with *K. pneumoniae* and *E. coli*, by Raman spectroscopic analysis (SpectraCell RA, River Diagnostics BV, Rotterdam, the Netherlands). Typing was performed according to the manufacturer’s instructions [13]. In short, isolates were inoculated on trypticase soy agar (TSA), incubated overnight at 35°C, and then checked for purity. Biomass (free-lying colonies) was collected from the TSA plates to fill a 1-μL loop, and suspended in 20 μL sterilized water. Twenty microliters of the suspension was inoculated and spread on a new TSA plate, and allowed to dry for 10 minutes. The plates were incubated at 35°C for 20 hours (±30 minutes). Using a 1-μL inoculation loop, the biomass was suspended in 10 μL of sterilized water, and centrifuged for 3 minutes at high speed (circa 10000*g*) to remove possible air bubbles. After removal of 4 μL supernatant, the pellet was resuspended, and 4 μL of suspension was pipetted into the indicated well of the MicroSlide and dried in an incubator for 20–30 minutes. Raman spectra were measured using the SpectraCell Bacterial Strain Analyzer. The similarity between pairs of spectra was calculated using the squared Pearson correlation coefficient (*R*^2^). The cutoff values used for the calculation of clusters of clonally related isolates were based on species-dependent criteria determined by the manufacturer.

#### Linkage and Cross-transmission

Epidemiological and microbiological linkages were used to identify possible cross-transmission events. Epidemiological linkage was defined as the presence of 2 patients with an overlapping stay in ICU. Microbiological linkage was defined as 2 patients being colonized with identical species belonging to a defined Raman cluster. Cross-transmission was defined as acquired colonization in a patient with negative cultures on admission, and both epidemiological and microbiological linkage to at least 1 other patient.

Cross-transmission rates were expressed as the number of cross-transmission events per 1000 patient-days at risk (DAR). DARs included all patient-days of uncolonized patients in ICU with at least 1 colonized patient in ICU. To account for colonization pressure, weighted days at risk (wDAR) were calculated by multiplying the DAR each day with the number of colonized patients during that day in the ICU. The overall averaged transmissibility ratio was determined using a mixed-effects Poisson model with number of transmissions as the outcome, species and log(wDAR) as fixed effects, and a random effect for hospital. Analyses were performed using SPSS version 20 and R version 2.15.1 software.

## RESULTS

For 11420 of the 14390 patients in the study, there was at least 1 culture result available (see Table 1 and Derde et al [7] for more details), of whom 637 patients were colonized with *E. coli* and 1184 with non-EcE. Admission prevalence was 3.8% for non-EcE species and 3.3% for *E. coli*. Of patients uncolonized at admission for non-EcE species, 7.4% had non-EcE in at least 1 subsequent culture. For *E. coli,* this was 2.6%. From 1046 of the 1184 patients colonized with non-EcE species, frozen isolates were available for further analysis and, of these, 777 (74.3%) were *K. pneumoniae*. The production of ESBL only was the predominant phenotype of ESC resistance (88.7%).

**Table 1.** Estimation of Transmission Parameters of Non–*Escherichia coli* Enterobacteriaceae and *E. coli* in 13 European Intensive Care Units Using a Random-Effects Model With No Effect of the Interventions

Parameter	Patients Included (n = 11420)	
	Non-EcE	*Escherichia coli*
No. of patients colonized at admission (%)	401 (3.8%)	356 (3.3%)
No. of patients with documented acquisition	783	281
Acquisition rate per 100 uncolonized admissions	7.4	2.6
Cross-transmission parameter *β*_*0*_, (95% CrI)	0.029 (.016–.049)	0.0078 (.0029–.016)
Single-admission reproduction number *R*_*A*_ (95% CrI)	0.17 (.094–.29)	0.047 (.018–.098)
Transmission parameter *α*_*0*_, (95% CrI)	0.0048 (.0022–.011)	0.0024 (.0013–.0039)
Relative transmission capacity of non–*E. coli* Enterobacteriaceae vs *E. coli* (*β*_*0*_^*non-EcE*^/*β*_*0*_^*E. coli*^) (95% CrI)	3.7 (1.4–11.3)	

Estimates are the values with the highest posterior probability density. Of 14390 patients, only the 11420 with at least 1 culture result were used in this analysis.

Abbreviation: CrI, credibility interval; non-EcE, non–*Escherichia coli* Enterobacteriaceae.

Maximum likelihood estimates for *α* and *β* differed between ICUs and between periods, ranging from 0.0001 to 0.013 and 0.0001 to 0.045 for *α* and *β* for *E. coli*, respectively, and from 0.0001 to 0.040 and 0.0001 to 0.105 for *α* and *β* for non-EcE, respectively (Supplementary Table 1). The random-effects analysis (assuming constant transmission parameters for all study periods in each ICU) yielded values with the highest posterior probability density and 95% credibility intervals (CrIs) of 0.0024 (.0013–.0039) and 0.0078 (.0029–.016) for *α*_*0*_ and *β*_*0*_ for *E. coli*, respectively, and 0.0048 (.0022–.011) and 0.029 (.016–.049) for *α*_*0*_ and *β*_*0*_ for non-EcE, respectively (Supplementary Figure 1 and Table 1). Using the observed mean length of stay of 6 days, these transmission parameters correspond to effective single-admission reproduction numbers of 0.047 (95% CrI, .018–.098) for *E. coli* and 0.17 (95% CrI, .094–.29) for non-EcE. The cross-transmission parameter is, therefore 3.7 (95% CrI, 1.4–11.3) times higher for non-EcE compared with *E. coli*. Higher relative cross-transmission parameters for non-EcE as compared to *E. coli* were also obtained when data were analyzed per study period, being 3.6 (95% CrI, 1.0–11.2), 2.4 (95% CI, .83–7.1), and 4.4 (95% CrI, 1.2–13.0) in periods 1, 2, and 3, respectively (Supplementary Table 2). The ratio was 4.3 (95% CrI, 2.1–7.6) when all study periods in all ICUs were considered to be independent from each other (see Supplementary Figure 1 and Supplementary Table 3 for more details).

### Raman Spectroscopy

In total, 1015 isolates (385 *E. coli* and 630 *K. pneumoniae*) from 877 patients from 4 ICUs (in Greece, France, Latvia, and Slovenia) were typed with Raman spectroscopic analysis. For *K. pneumoniae*, 173 patients (174 isolates) were colonized on admission (4.1% admission prevalence), and 449 acquired carriage (456 isolates, 10.7% acquisition rate). For *E. coli*, 214 patients (215 isolates) were colonized on admission (5.1%), and 169 acquired carriage (170 isolates, 4.0%). Transmission rates ranged across ICUs from 1.66 to 29.74/1000 DAR for *K. pneumoniae* and from 0 to 3.31/1000 DAR for *E. coli* (Supplementary Table 4). Assuming equal transmissibility ratios in hospitals and using the wDAR, the combined transmissibility ratio is 5.0:1 (95% CI for the population average, 3.6–7.1:1). The estimated transmissibility ratio in these 4 ICUs based on modeling with random effects was 6.1 (95% CrI, 1.3:1–35.0:1). When we performed our mathematical model with a random-effects analysis for these 4 hospitals only, we found as value for the transmissibility ratio with the highest posterior probability density: 6.1 (95% CrI, .69–58.6).

## DISCUSSION

Based on extensive microbiological surveillance in 13 ICUs during a 24-month period and mathematical modeling, the estimated relative cross-transmission capacity of non-EcE (mainly consisting of *K. pneumoniae*, but also *Enterobacter*, *Serratia*, and *Citrobacter* species) was found to be 3.7 times higher than that of *E. coli.* Importantly, external factors influencing transmission could be considered equal during the study period for all species. The estimates in a subset of 4 ICUs using bacterial typing by the Raman spectroscopy analysis were very similar to the estimates of the random-effects model based on the same 4 ICUs. The per-admission reproduction numbers were 0.17 for non-EcE and 0.047 for *E. coli*.

This was a post hoc analysis of a large international prospective study, and, therefore, inevitably has study limitations. The surveillance method as used may have resulted in misclassification of some patients. Only the first isolate of each morphotype identified on chromogenic media was selected for species determination. Therefore, carriage with *K. pneumoniae* could have been missed in patients colonized with either *Enterobacter*, *Serratia*, or *Citrobacter* species, as no further isolates were harvested and tested. Furthermore, for confirmation of our results, we used the high-throughput typing method by the Raman spectroscopy (Supplementary Material 3) for *K. pneumoniae* and *E. coli* isolates of 4 ICUs. Although validated for typing antibiotic-resistant Enterobacteriaceae [14], whole-genome sequencing might have provided more granularity and, thereby, more accurate estimates of transmission parameters.

When using mathematical modeling it is inevitable to make assumptions. Naturally, transmission will differ between ICUs, but the study was underpowered for reliably estimating transmission ratios per ICU. However, by using a random-effects model, differences between ICUs were taken into account. Furthermore, we assumed that the sensitivity and specificity of the microbiologic tests was 100%, mainly to reduce the computational burden. However, this assumption affected both *E. coli* and non-EcE, and it is, therefore, unlikely that it will impact the estimated relative transmission capacity of *E. coli* and non-EcE.

The higher estimates for these 4 ICUs (although not statistically significant) compared to the analysis for all 13 ICUs could be the result of selection bias. As we required that at least 25 patients were colonized with *K. pneumoniae* and *E. coli*, we might have selected for ICUs with unnoticed *K. pneumoniae* outbreak during the study period.

Our findings are in line with previous results suggesting that in-hospital transmission was higher for ESBL-producing *K. pneumoniae* than for ESBL-producing *E. coli*, although the observed transmission rates of 13.9 and 5.6 cases per 1000 exposure-days for *K. pneumoniae* and *E. coli*, respectively, were based on only 2 and 4 transmission events in a hospital-wide setting, respectively [15]. In another study, patient-to-patient transfer, based on epidemiological linkage and pulsed-field gel electrophoresis typing, was observed in 14 of 27 (52%) acquisitions with ESBL-producing *K. pneumoniae* and in 3 of 23 (13%) acquisitions with ESBL-producing *E. coli* [13, 16]. Because of these small numbers, it was not possible to quantify the amount of and uncertainty in transmission capacity of the different species in those studies.

Our study estimates that per-admission effective reproduction numbers for non-EcE and *E. coli* were well below 1 (0.17 and 0.047, respectively), suggesting that outbreaks typically remain small with current infection control policies. These findings support the observation that treating carriers of these bacteria in isolation, which was the cluster-randomized intervention in this study, failed to reduce the prevalence of carriage in the ICUs. In fact, our findings also suggest that the implementation of universal chlorhexidine body washing and improving hand hygiene adherence from 52% to 77% did not reduce the transmission capacity of these bacteria. Yet, our estimated *R*_*A*_ of 0.17 for non-EcE should not be interpreted as evidence for ineffectiveness of isolation measures for *K. pneumoniae*. First, isolation will only be effective if there is cross-transmission. In our setting, with low transmission rates, the potential gain of isolation is limited. Yet, in settings with high rates of cross-transmission, isolation may be effective. Note that in high-endemicity settings, isolation will not be effective if the high-endemicity levels are primarily caused by a high admission prevalence of ESC-Enterobacteriaceae carriage, for example, due to extramural reservoirs. Second, the estimate is for the group of bacteria, and the individual estimate for *K. pneumoniae* could be higher. Moreover, as compared to *E. coli*, *K. pneumoniae* is more frequently also resistant to carbapenem antibiotics, providing further arguments to prevent cross-transmission.

In conclusion, the analysis of extensive longitudinal carriage data from 13 European ICUs demonstrated that the transmission rate of non-EcE (mainly consisting of *K. pneumoniae*) is 3.7 times higher than that of *E. coli.* If problems emerge (eg, outbreaks of colistin-resistant Enterobacteriaceae), more measures are needed to control a *K. pneumoniae* outbreak than are needed to control an *E. coli* outbreak.

## Supplementary Data

Supplementary materials are available at *Clinical Infectious Diseases* online. Consisting of data provided by the authors to benefit the reader, the posted materials are not copyedited and are the sole responsibility of the authors, so questions or comments should be addressed to the corresponding author.

## Supplementary Data

Supplementary materials are available at *Clinical Infectious Diseases* online. Consisting of data provided by the authors to benefit the reader, the posted materials are not copyedited and are the sole responsibility of the authors, so questions or comments should be addressed to the corresponding author.

## Supplementary Material

Supplementary Material

## Notes

***Financial support.*** This work and M. J. D. D., L. P. G. D., and T. G. were supported by the European Commission under the Life Science Health Priority of the Sixth Framework Program (MOSAR network contract LSHP-CT-2007-037941). M. J. M. B. was supported by the Netherlands Organization of Scientific Research (VICI NWO grant number 918.76.611). M. C. J. B. was supported by the European Community (RGNOSIS Integrated project [FP7/2007-2013] under grant agreement number 282512.). M. G has received funding from Curetis GmbH for consulting and from bioMérieux for travel expenses.

***Potential conflicts of interest.*** All authors: No reported conflicts. All authors have submitted the ICMJE Form for Disclosure of Potential Conflicts of Interest. Conflicts that the editors consider relevant to the content of the manuscript have been disclosed.
