# Supplementary material for: The Transmissibility of Antibiotic-Resistant Enterobacteriaceae in Intensive Care Units
Source: Clin Infect Dis. 2017 Sep 15;66(4):489–93. doi: 10.1093/cid/cix825 (PMC5850446; doi:10.1093/cid/cix825)
Supplement: Supplementary Material [file cix825_suppl_supplementary_material.docx]

**Supplementary material 1**

From the MOSAR ICU trial [6] we have data on the admission and discharge day of patients in 13 ICU in Europe and data on their colonization status at certain points in time (day of screening and result of screening), i.e., at admission and afterwards twice per week. The study consisted of 3 phases, which we initially analyze separately.

We assumed that the probability to acquire colonization equals$\alpha+\beta\frac{I(t)}{N(t)}$ where *α* represents the risk due to routes which do not depend on the number of colonized patients present in the ICU, the so-called endogenous route, and $\beta\frac{I(t)}{N(t)}$ describes the rate of cross-transmission where *I(t)* is the number of colonized patients in the ICU at time *t* and *N(t)* is the total number of patients present in ICU at time *t*. The parameter *β* represents the effective transmissibility of the bacteria in the ICU, taking infection control measures like hand hygiene into account. We want to know what the probability is to observe the data as were observed in one of the ICUs if the transmission parameters were (*α*,*β*). An existing algorithm, written in C++, can be used [5] to determine the likelihood of the data for given values of the parameters *𝛼* and *𝛽.* Below we shortly describe the algorithm.

We divide the stay of each patient into at most three periods:

- The period when the patient is known to be uncolonized, i.e., from admission to the last negative test;
- The period when the colonization status of a patient is unknown, i.e., from the last negative test to first positive test (if there was such) or from the last negative test to discharge;
- The period when the patient is known to be colonized, i.e., from first positive test to discharge.

Note that this distinction can be made because we assume that colonization is persistent during ICU stay and the test results are 100% reliable. If we would know the exact moment of colonization of each patient, calculation of the likelihood is easy^5^. Our algorithm calculates the likelihood of the observed culture results by a (weighted) summation over all possible moments of acquisition of all patients.

The likelihood was calculated on a grid of step size 0.005 for *α* and 0.0005 for *β*. In this way for each ICU for each period we obtained the likelihood of the data for each point of the grid.
For values close to the maximum likelihood estimates of *α* and *β* we refined our grid to obtain more precise estimates. The likelihood for parameters *α* and *β* between grid points was determined by linear interpolation.

**Supplementary material 2: Random effects model**

To reflect that the transmission parameters may differ between ICUs we assume that the transmission parameters of a certain ICU are realizations of two independent folded normal distributions, i.e., $\alpha\sim N(\alpha_{0},\sigma_{\alpha})$ and $\beta\sim N(\beta_{0},\sigma_{\beta})$. Formally, this implies a switch to a Bayesian perspective. We assume non-informative (improper) priors for $\alpha_{0},\sigma_{\alpha},\beta_{0}\mathrm{and}\sigma_{\beta}$

Case 1: We assume here, for simplification, that the transmission parameters of a certain bacteria in a certain ICU in the three periods (baseline and 2 intervention periods) are independent of each other (different bundle, treatment during different periods of the trial).

We calculate the posterior distribution *H* for each point (*α_0_*,*β_0_*) on the grid by the formula:

$H\left( {icu,phase,\alpha}_{0},\sigma_{\alpha},\beta_{0},\sigma_{\beta} \right)\sim\frac{1}{\sigma_{\alpha}\sqrt{2\pi}}\frac{1}{\sigma_{\beta}\sqrt{2\pi}}\iint e^{-\frac{\left( \alpha-\alpha_{0} \right)^{2}}{2{\sigma_{\alpha}}^{2}}}e^{-\frac{\left( \beta-\beta_{0} \right)^{2}}{2{\sigma_{\beta}}^{2}}}L\left( icu,phase,\alpha,\beta\right) d\alpha d\beta$,

where $L(icu,phase,\alpha,\beta)$ is the likelihood of the observed data during the trial if the transmission parameters are *α* and *β* for endogenous and cross-transmission respectively (see Supplementary Material 1).

The overall posterior distribution is obtained by the product over all ICUs and all phases of the posterior distribution per ICU and per phase, i.e., $H_{all}\left( \alpha_{0},\sigma_{\alpha},\beta_{0},\sigma_{\beta} \right)\sim\prod_{all icu, all phases} H\left( icu{,phase, \alpha}_{0},\sigma_{\alpha},\beta_{0},\sigma_{\beta} \right)$

Case 2: Despite different measures during the three phases, no significant effect of the intervention on the spread of Gram-negative bacteria was found [Derde et al]. Therefore, in our main analysis, we assume that the transmission parameters in phase 2 and 3 were the same as in period 1.

Now the formula for the posterior distribution per ICU is:

$$H\left( {icu,\alpha}_{0},\sigma_{\alpha},\beta_{0},\sigma_{\beta} \right)\sim\frac{1}{\sigma_{\alpha}\sqrt{2\pi}}\frac{1}{\sigma_{\beta}\sqrt{2\pi}}\iint e^{-\frac{\left( \alpha-\alpha_{0} \right)^{2}}{2{\sigma_{\alpha}}^{2}}}e^{-\frac{\left( \beta-\beta_{0} \right)^{2}}{2{\sigma_{\beta}}^{2}}}\cdot\cdot L\left( icu,phase=1,\alpha,\beta\right)L\left( icu,phase=2,\alpha,\beta\right)L\left( icu,phase=3,\alpha,\beta\right) d\alpha d\beta$$

The overall posterior distribution is obtained by the product over all ICUs of the posterior distribution per ICU, i.e., $H_{all}(\alpha_{0},\sigma_{\alpha},\beta_{0},\sigma_{\beta})\sim\prod_{all icu} H\left( icu{, \alpha}_{0},\sigma_{\alpha},\beta_{0},\sigma_{\beta} \right)$

**Supplementary material 3: Effects assumptions perfect sensitivity of the test**

A formal investigation of how the assumption of perfect sensitivity and specificity of the test impact our estimates would be a manuscript on its own. To try to shed some light, we have run 10 simulations of a ward of 10 beds for a period of 1000 days with the parameter values for non-EcE observed in this study ($\alpha$= 0.0048, *β* =0.029 and the admission prevalence is 3.8%, with cultures performed on admission and afterwards twice per week and with a mean length of stay of 6 days). In our simulation we assumed a sensitivity of the test of 80% and a specificity of 100%. For each simulated data set we subsequently estimated the transmission parameter β for two cases: 1) if we assume a 100% sensitivity of the test and 2) if we estimate the sensitivity simultaneously with the estimate of the parameter *β* (we used an Bayesian approach described in Worby et al. Am J Epidemiol (2013) 177 (11): 1306-1313). In all 10 simulations, the estimate of the sensitivity was close to the true value of 80%. The median values for *β* was, on average, 4.5% lower with an assumed sensitivity of 100% as compared to the value of *β* obtained when the sensitivity was estimated simultaneously. The range of the ratio of the two estimates was (0.88-1.17), so the maximum deviation observed was 17%, which is also the maximum error in the estimate of *R_A_*. This suggest that the errors made by assuming a test with perfect sensitivity and specificity are moderately low.

The relative transmission capacity of *E.coli* and non-EcE does depend even less on the sensitivity and specificity of the test, as long as the sensitivity and specificity for E. coli and non-EcE are comparable. The reason is that the estimates of the *R_A_*-value for *E. coli* and for non-EcE are calculated in the same way, and the systematic errors in the estimate are therefore expected to be of the same relatively size , which implies that they are cancelled out when taken the ratio of the two *R_A_*-values

|  | *E. coli* | Non-EcE |
| --- | --- | --- |
| No effect | 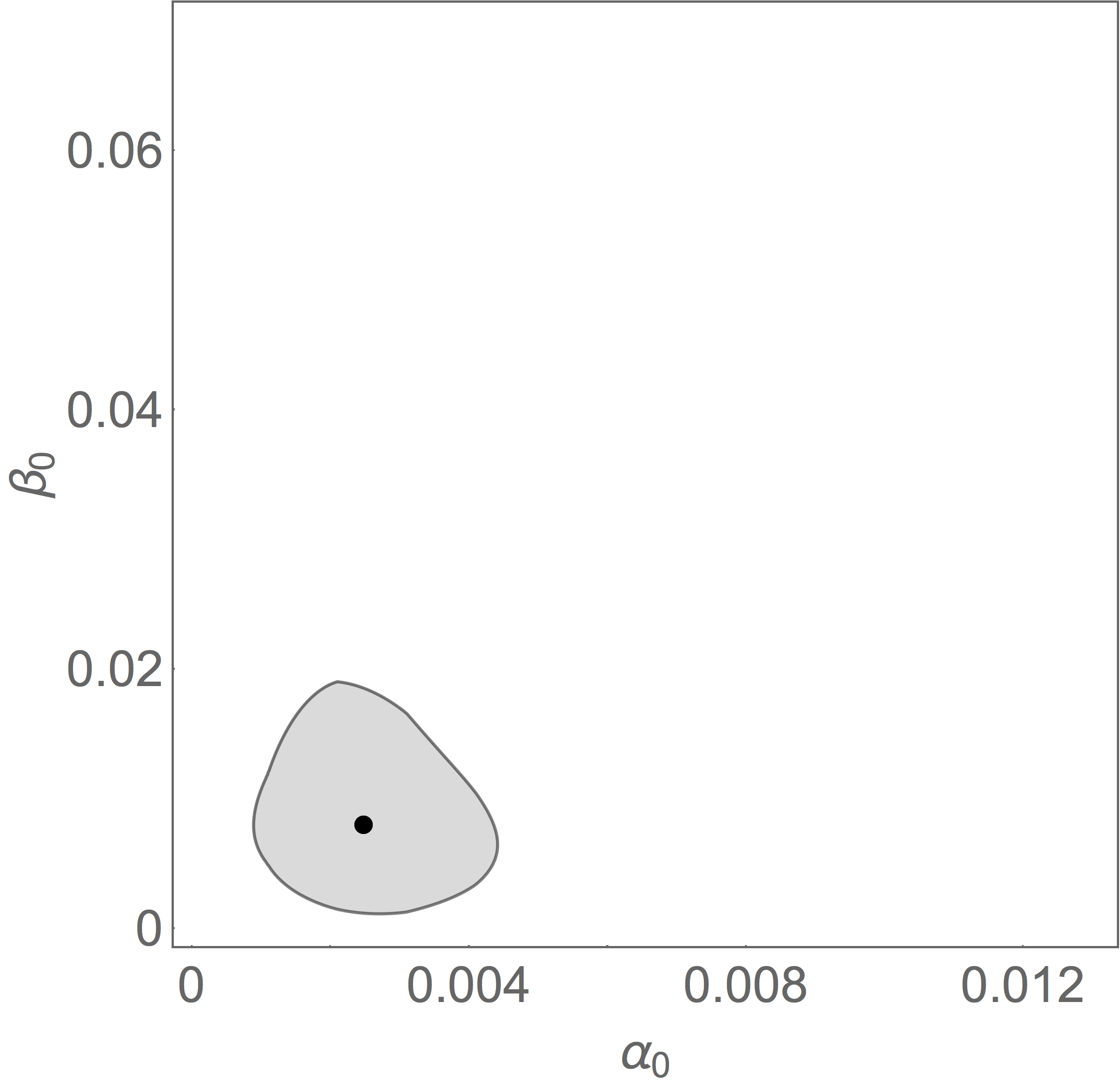 | 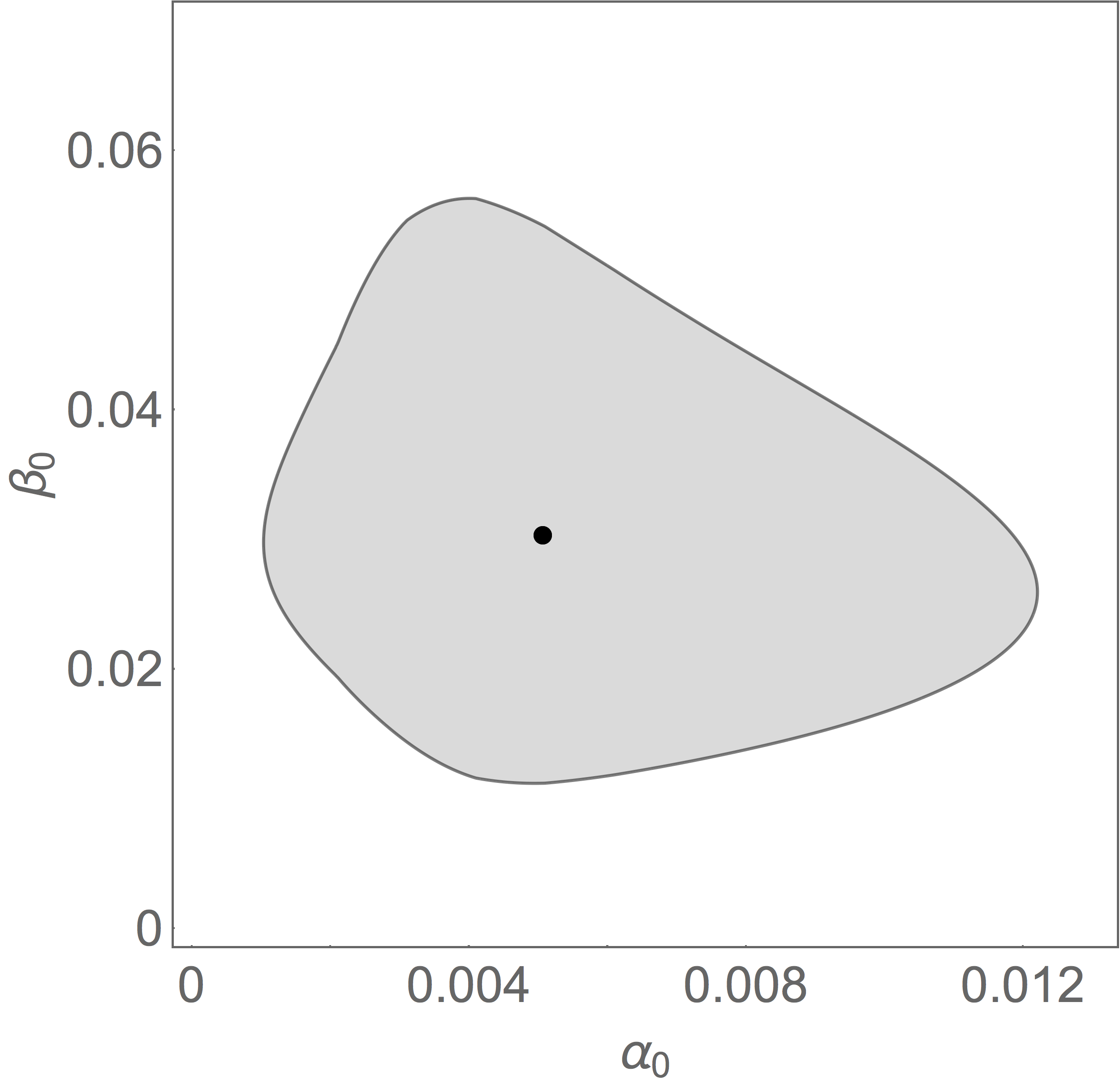 |
| independent | 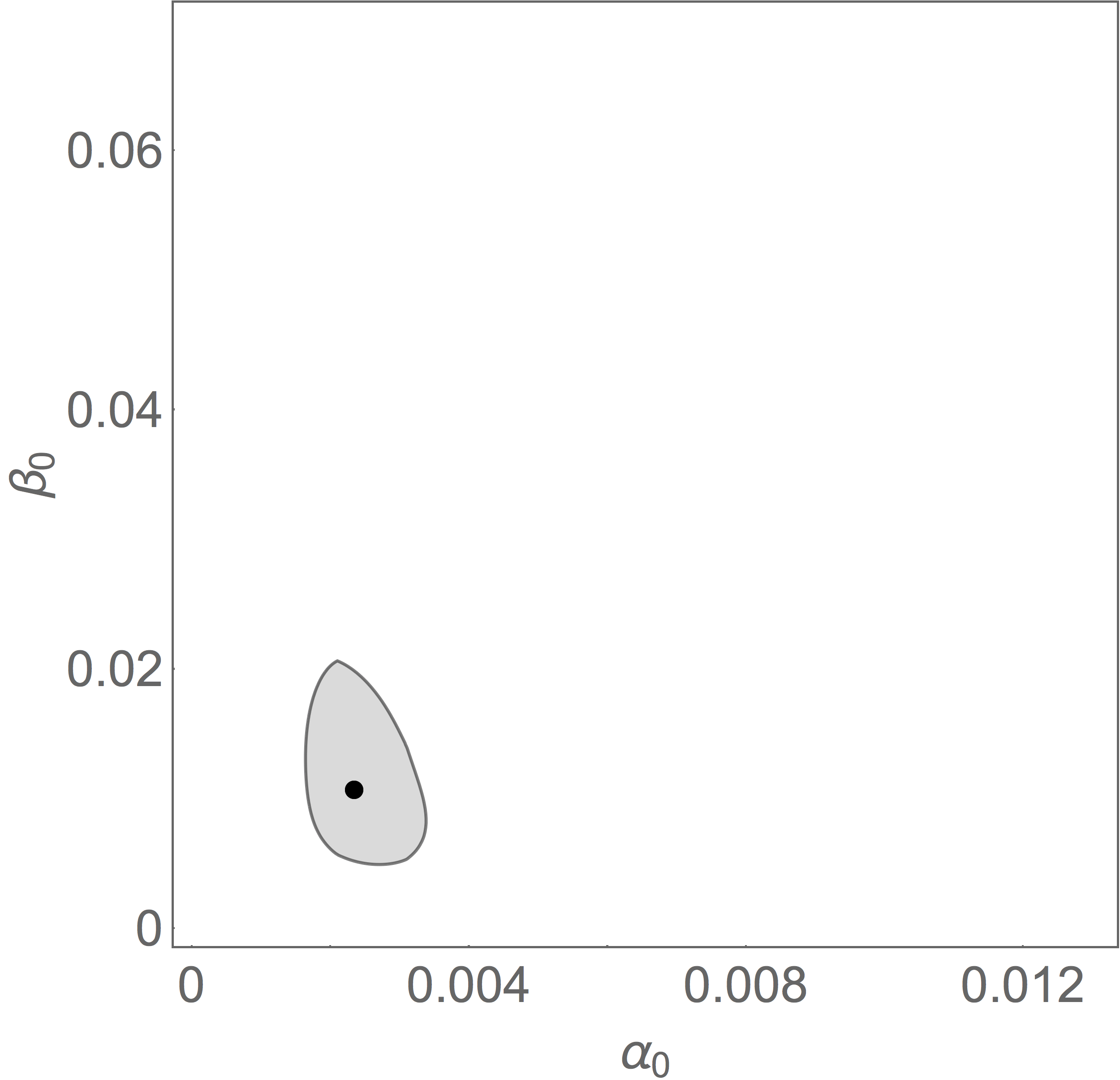 | 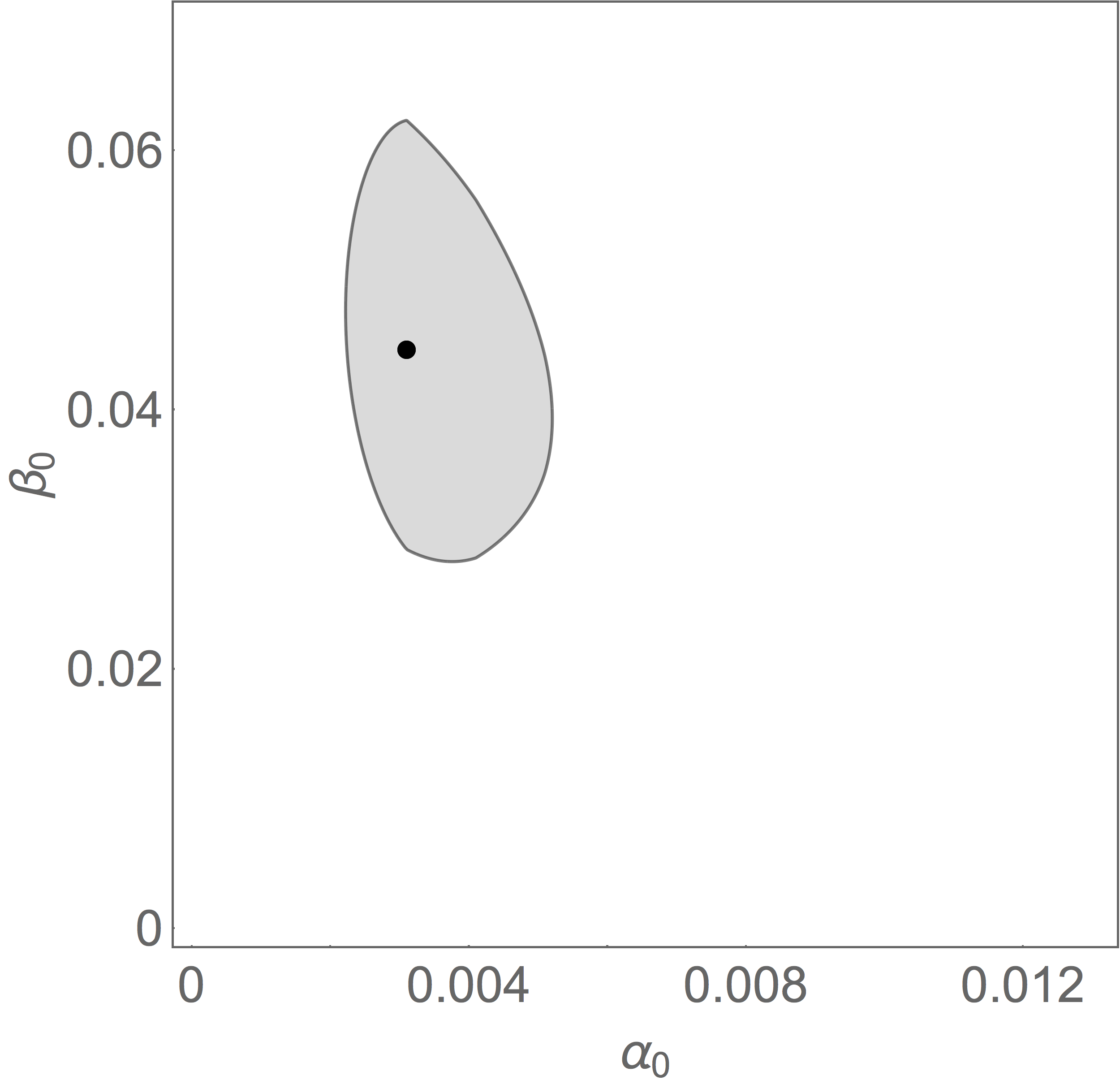 |

Figure S1. Estimates of the transmission parameters (*α_0_*, *β_0_*) and 95% credibility areas for *E. coli* (left) and non-*E. coli* Enterobacteriaceae (non-EcE) (right) using a random effect model with the assumption that the intervention had no effect the transmission parameters (upper panels) and with the assumption that the transmission parameters in each period were independent (lower panel). The transmission parameter of the endogenous route is on the horizontal axis and the cross-transmission parameter is on the vertical axis.

Table S1. Estimation of transmission parameters (*α,β*) and 95% confidence interval of non-*E. coli* Enterobacteriaceae (non-EcE) and *E. coli* in 13 European intensive care units per ICU per period.

| phase |  | 1 | | | 2 | | 3 | |
| --- | --- | --- | --- | --- | --- | --- | --- | --- |
|  |  | α | | β | α | β | α | β |
| ICU |  |  | |  |  |  |  |  |
| 1 | non-EcE | 0.0046 | 0.0251 | | 0.0151 | 0.0001 | 0.0051 | 0.0051 |
|  |  | (0.0006-0.0126) | (0.0001-0.0551) | | (0.0031-0.0201) | (0.0001-0.0401) | (0.0016-0.0076) | (0.0001-0.0351) |
|  | *E. coli* | 0.0011 | 0.0001 | | 0.0011 | 0.0001 | 0.0031 | 0.0051 |
|  |  | (0.0006-0.0026) | (0.0001-0.0201) | | (0.0006-0.0026) | (0.0001-0.0351) | (0.0016-0.0046) | (0.0001-0.0351) |
| 2 | non-EcE | 0.0001 | 0.1001 | | 0.0011 | 0.0001 | 0.0006 | 0.0001 |
|  |  | (0.0001-0.0021) | (0.0051-0.2951) | | (0.0001-0.0051) | (0.0001-0.2951) | (0.0001-0.0016) | (0.0001-0.1601) |
|  | *E. coli* | 0.0016 | 0.0451 | | 0.0016 | 0.0301 | 0.0036 | 0.0001 |
|  |  | (0.0001-0.0081) | (0.0001-0.1251) | | (0.0001-0.0061) | (0.0001-0.1351) | (0.0021-0.0061) | (0.0001-0.0301) |
| 3 | non-EcE | 0.0121 | 0.0001 | | 0.0026 | 0.0251 | 0.0016 | 0.0201 |
|  |  | (0.0051-0.0201) | (0.0001-0.0701) | | (0.0006-0.0091) | (0.0001-0.1051) | (0.0006-0.0041) | (0.0001-0.0601) |
|  | *E. coli* | 0.0001 | 0.0251 | | 0.0001 | 0.0251 | 0.0011 | 0.0301 |
|  |  | (0.0001-0.0026) | (0.0051-0.0801) | | (0.0001-0.0026) | (0.0001-0.1101) | (0.0006-0.0031) | (0.0051-0.0701) |
| 4 | non-EcE | 0.0011 | 0.0351 | | 0.0026 | 0.0101 | 0.0016 | 0.0301 |
|  |  | (0.0001-0.0036) | (0.0001-0.1751) | | (0.0006-0.0091) | (0.0001-0.0551) | (0.0006-0.0046) | (0.0051-0.0651) |
|  | *E. coli* | 0.0006 | 0.0001 | | 0.0016 | 0.0101 | 0.0026 | 0.0001 |
|  |  | (0.0001-0.0031) | (0.0001-0.0551) | | (0.0001-0.0066) | (0.0001-0.0851) | (0.0011-0.0056) | (0.0001-0.0301) |
| 5 | non-EcE | 0.0086 | 0.0351 | | 0.0076 | 0.0201 | 0.0051 | 0.0201 |
|  |  | (0.0026-0.0176) | (0.0001-0.0851) | | (0.0026-0.0151) | (0.0001-0.0651) | (0.0011-0.0101) | (0.0001-0.0501) |
|  | *E. coli* | 0.0046 | 0.0001 | | 0.0041 | 0.0001 | 0.0056 | 0.0001 |
|  |  | (0.0016-0.0076) | (0.0001-0.0401) | | (0.0016-0.0071) | (0.0001-0.0301) | (0.0036-0.0071) | (0.0001-0.0101) |
| 6 | non-EcE | 0.0401 | 0.0251 | | 0.0276 | 0.0401 | 0.0106 | 0.0201 |
|  |  | (0.0056-0.0401) | (0.0001-0.1101) | | (0.0061-0.0401) | (0.0001-0.1051) | (0.0031-0.0236) | (0.0001-0.0501) |
|  | *E. coli* | 0.0001 | 0.0001 | | 0.0006 | 0.0001 | 0.0001 | 0.0001 |
|  |  | (0.0001-0.0021) | (0.0001-0.1451) | | (0.0001-0.0031) | (0.0001-0.2301) | (0.0001-0.0006) | (0.0001-0.1051) |
| 7 | non-EcE | 0.0056 | 0.0301 | | 0.0136 | 0.0001 | 0.0091 | 0.0751 |
|  |  | (0.0001-0.0191) | (0.0001-0.0701) | | (0.0066-0.0241) | (0.0001-0.0351) | (0.0001-0.0401) | (0.0001-0.1251) |
|  | *E. coli* | 0.0041 | 0.0001 | | 0.0026 | 0.0001 | 0.0036 | 0.0001 |
|  |  | (0.0001-0.0091) | (0.0001-0.0751) | | (0.0006-0.0081) | (0.0001-0.0451) | (0.0016-0.0066) | (0.0001-0.0351) |
| 8 | non-EcE | 0.0006 | 0.0001 | | 0.0001 | 0.0101 | 0.0016 | 0.0001 |
|  |  | (0.0001-0.0016) | (0.0001-0.1251) | | (0.0001-0.0016) | (0.0001-0.0501) | (0.0006-0.0026) | (0.0001-0.0201) |
|  | *E. coli* | 0.0006 | 0.0001 | | 0.0006 | 0.0001 | 0.0021 | 0.0001 |
|  |  | (0.0001-0.0016) | (0.0001-0.1801) | | (0.0001-0.0016) | (0.0001-0.2951) | (0.0016-0.0036) | (0.0001-0.0101) |
| 9 | non-EcE | 0.0016 | 0.0001 | | 0.0021 | 0.0351 | 0.0011 | 0.0001 |
|  |  | (0.0006-0.0041) | (0.0001-0.1851) | | (0.0006-0.0056) | (0.0001-0.1401) | (0.0006-0.0021) | (0.0001-0.1151) |
|  | *E. coli* | 0.0031 | 0.0001 | | 0.0021 | 0.0001 | 0.0011 | 0.0151 |
|  |  | (0.0011-0.0066) | (0.0001-0.0501) | | (0.0006-0.0056) | (0.0001-0.0401) | (0.0006-0.0026) | (0.0001-0.1051) |
| 10 | non-EcE | 0.0401 | 0.0051 | | 0.0401 | 0.0651 | 0.0136 | 0.1051 |
|  |  | (0.0186-0.0401) | (0.0001-0.0601) | | (0.0121-0.0401) | (0.0401-0.1251) | (0.0001-0.0366) | (0.0501-0.1501) |
|  | *E. coli* | 0.0126 | 0.0001 | | 0.0046 | 0.0251 | 0.0071 | 0.0251 |
|  |  | (0.0066-0.0176) | (0.0001-0.0401) | | (0.0006-0.0126) | (0.0001-0.0601) | (.0036-0.0116) | (0.0051-0.0451) |
| 11 | non-EcE | 0.0026 | 0.0251 | | 0.0036 | 0.0551 | 0.0041 | 0.0101 |
|  |  | (0.0006-0.0081) | (0.0001-0.0801) | | (0.0011-0.0076) | (0.0001-0.1851) | (0.0021-0.0071) | (0.0001-0.0301) |
|  | *E. coli* | 0.0016 | 0.0301 | | 0.0016 | 0.0401 | 0.0021 | 0.0001 |
|  |  | (0.0001-0.0061) | (0.0001-0.0801) | | (0.0006-0.0051) | (0.0001-0.1701) | (0.0011-0.0036) | (0.0001-0.0101) |
| 12 | non-EcE | 0.0026 | 0.0001 | | 0.0026 | 0.0351 | 0.0061 | 0.0301 |
|  |  | (0.0006-0.0061) | (0.0001-0.0301) | | (0.0006-0.0086) | (0.0001-0.1401) | (0.0026-0.0116) | (.0001-0.1051) |
|  | *E. coli* | 0.0041 | 0.0001 | | 0.0021 | 0.0001 | 0.0011 | 0.0301 |
|  |  | (0.0016-0.0091) | (0.0001-0.0251) | | (0.0006-0.0066) | (0.0001-0.0301) | (0.0001-0.0036) | (0.0001-0.1351) |
| 13 | non-EcE | 0.0036 | 0.0001 | | 0.0061 | 0.0001 | 0.0011 | 0.0001 |
|  |  | (0.0011-0.0091) | (0.0001-0.0651) | | (0.0026-0.0116) | (0.0001-0.0751) | (0.0006-0.0036) | (0.0001-0.0501) |
|  | *E. coli* | 0.0031 | 0.0301 | | 0.0001 | 0.0001 | 0.0001 | 0.0001 |
|  |  | (0.0006-0.0086) | (0.0001-0.2451) | | (0.0001-0.0016) | (0.0001-0.2951) | (0.0001-0.0011) | (0.0001-0.0751) |

Table S2. Estimation of transmission parameters of non-*E. coli* Enterobacteriaceae (non-EcE) and *E. coli* in 13 European intensive care units per phase using a random effects model.

|  | **Period 1**  **(0.5 year)** | | **Period 2**  **(0.5 year)** | | | **Period 3**  **(1 year)** | |  |
| --- | --- | --- | --- | --- | --- | --- | --- | --- |
|  | non-EcE | *E. coli* | | non-EcE | *E. coli* | non-EcE | *E. coli* |  |
| Number of patients | 2819 | | | 2523 | | 6078 | | |
| Patients colonized at admission | 102 | 91 | | 94 | 57 | 205 | 208 | |
| Acquisitions | 181 | 69 | | 213 | 47 | 389 | 165 | |
| Cross transmission parameter *β_0_*, (95% CI) | 0.041  (0.020-0.067) | 0.011 (0.0041-0.031) | | 0.041 (0.019-0.070) | 0.017 (0.0066-0.020) | 0.040 (0.017-0.067) | 0.0093 (0.0034-  0.022) | |
| Transmission parameter *α_0_* (endogenous route), (95% CI) | 0.0035 (0.0019-0.0095) | 0.0026 (0.0014-0.0045) | | 0.0048 (0.0027-0.0099) | 0.0016 (0.0008-0.0035) | 0.0025 (0.0015-0.0047) | 0.0025 (0.0014-0.0039) | |
| Single admission reproduction number *R_A_* (95% CI) | 0.25 (0.12-0.40) | 0.068 (0.024-0.18) | | 0.25 (0.11-0.42) | 0.10  (0.040-0.21) | 0.24  (0.10-0.40) | 0.056  (0.021- 0.13) | |
| Relative transmission capacity of non-EcE versus *E. coli*  (*β_0_*^non-EcE^/*β_0_*^E. coli^) (95% CI) | 3.6 (1.0-11.2) | | 2.4 (0.83-7.1) | | | 4.4 (1.2-13.0) | |  |

CI: credibility interval. Estimates are the values with the highest posterior posterior probability density.

Table S3. Estimates of the transmission parameters of non-*E. coli* Enterobacteriaceae (non-EcE) and *E. coli* in 13 European intensive care units using a random effects model while we assumed that the transmission parameters in different periods are independent.

|  | | Patients included (n=11,420) | | | |
| --- | --- | --- | --- | --- | --- |
|  |  | non-EcE | | *E. coli* | |
| Patients colonized at admission (%) | | 401 (3.5%) | | 356 (3.1%) | |
| Acquisitions | | 783 | | 281 | |
| Acquisition rate/100 uncolonized admissions | 7.4 | | 2.6 | |  |
| Cross transmission parameter *β_0_*, (95% CI) | | 0.044 (0.031-0.058) | | 0.010 (0.0061-0.019) | |
| Transmission parameter *α_0_* (endogenous route),  (95% CI) | | 0.0031 (0.0025-0.0049) | | 0.0023 (0.0018-0.0032) | |
| Single admission reproduction number *R_A_* (95% CI) | 0.26 (0.19-0.35) | | 0.061 (0.037-0.11) | |  |
| The relative transmission capacity of non-EcE versus *E. coli* (*β_0_^non-EcE^*/*β_0_^E. coli^)* (95% CI) | | 4.3 (2.1-7.6) | | | |

CI: credibility interval. Estimates are the values with the highest posterior probability density.

Table S.4. Transmissibility of *K. pneumoniae* and *E. coli* isolates in four European intensive care units using Raman spectroscopy.

|  | **ICU 1**  **(N=779)** | | **ICU 2**  **(N=1532)** | | **ICU 3**  **(N=625)** | | **ICU 4**  **(N=1309)** | |
| --- | --- | --- | --- | --- | --- | --- | --- | --- |
|  | KP | EC | KP | EC | KP | EC | KP | EC |
| On admission prevalence^a^ | 6.4 | 1.5 | 4.9 | 7.2 | 8.6 | 3.7 | 8.3 | 5.7 |
| Number of patients with isolate(s) available^b^ | 72 | 33 | 112 | 159 | 76 | 29 | 362 | 162 |
| Number of patients colonized on admission | 23 | 13 | 49 | 112 | 22 | 18 | 79 | 70 |
| Number of patients acquiring colonization | 49 | 20 | 63 | 47 | 54 | 10 | 283 | 92 |
| Number of transmissions | 29 | 0 | 19 | 3 | 28 | 0 | 246 | 33 |
| DAR | 11896 | 10184 | 11440 | 11764 | 4373 | 2482 | 8271 | 9974 |
| Transmissions/ 10000 DAR | 2.44 | 0 | 1.66 | 0.26 | 6.40 | 0 | 29.74 | 3.31 |
| wDAR | 31937 | 14571 | 23058 | 26109 | 9351 | 3369 | 49567 | 27354 |
| Transmissions/ 10000 wDAR | 0.91 | 0 | 0.82 | 0.11 | 2.99 | 0 | 4.96 | 1.21 |
| Number of extra transmissions using 7-day window | 0 | 0 | 4 | 1 | 4 | 0 | 6 | 7 |

DAR: Days at risk. EC: *Escherichia coli*. ICU: Intensive care unit. KP: *Klebsiella pneumoniae*. wDAR: weighted days at risk. ^a^ On admission prevalence of *E. coli* and non-*E. coli* Enterobacteriaceae . ^b^ For 10 patients 2 isolates were available (for EC 0, 1, 0 and 1 patients and for KP 2, 1, 1 and 4 patients for ICUs 1, 2, 3 and 4 respectively).
